# White Matter Integrity of the Corpus Callosum and Dissociative Tendencies: A Cross-Sectional Analysis

**DOI:** 10.1192/j.eurpsy.2026.11625

**Published:** 2026-07-31

**Authors:** P. Podwalski, B. Dawidowski, L. Franczak, B. Misiak, J. Samochowiec

**Affiliations:** 1 Department of Psychiatry, Pomeranian Medical University, Szczecin; 2 Department of Psychiatry, Wroclaw Medical University, Wrocław, Poland

## Abstract

**Introduction:**

Dissociative symptoms are common in clinical populations exposed to trauma and are hypothesized to involve disrupted interhemispheric connectivity. Diffusion tensor imaging (DTI) studies have implicated alterations in the corpus callosum (CC), but evidence remains inconsistent. We examined whether fractional anisotropy (FA) in CC regions is associated with self-reported dissociative tendencies.

**Objectives:**

The objective of this study was to examine whether FA in the CC —both globally and within a specific segment—is associated with dissociative tendencies. Advanced statistical procedures and sensitivity analyses with covariates were applied to assess the reliability of effects and to determine the extent to which callosal microstructure explains variability in dissociative symptoms.

**Methods:**

Eighty-two adults (clinical and healthy controls) completed the Dissociative Experiences Scale (DEST). Mean FA across the corpus callosum (CC_MEAN_DTI) and segment 6 (CC_6_MEAN_DTI) were extracted from DTI. Linear regressions were performed with DEST as the dependent variable, after Box–Cox transformation selected by AIC (λ = –1). Assumption checks guided inference: in case of heteroscedasticity or non-normal residuals, heteroskedasticity-robust SEs and bootstrap (wild or residual, B=2000) were applied. Multiple testing was controlled per marker using Benjamini–Hochberg FDR. Sensitivity analyses included bootstrap stability and stepwise models with covariates (Group, BMI, education).

**Results:**

Both FA measures showed negative associations with dissociative tendencies (β ≈ –0.67 for CC_MEAN_DTI; β ≈ –0.76 for CC_6_MEAN_DTI), suggesting lower FA might correspond to higher dissociation. However, after robust bootstrapping, neither effect was statistically significant (p ≈ 0.46; FDR-adjusted p ≈ 0.46). Variance explained was modest (R² ~6–8%). Stepwise models including group status and covariates further attenuated the associations (β reduced by ~80%), indicating that apparent links were largely confounded. Bootstrap stability was moderate, but confidence intervals overlapped zero when tested adaptively.

**Conclusions:**

This study did not find statistically reliable associations between FA in the corpus callosum and dissociative tendencies. Although trends were in the hypothesized direction (reduced CC integrity linked to higher dissociation), effects were small, unstable, and largely explained by other factors. Results highlight the need for larger samples and longitudinal designs to clarify whether callosal microstructure contributes to dissociative psychopathology.

**Disclosure of Interest:**

None Declared

